# Current status, challenges and future development of self-service appointment platforms in hospital radiology departments in China: a narrative review

**DOI:** 10.3389/fdgth.2026.1940548

**Published:** 2026-09-24

**Authors:** Peng-wei Shu, Ke-ke Lu, Shi-hao Xie, Zi-qiao Lei, Qing Fu

**Affiliations:** 1 Department of Radiology, Union Hospital, Tongji Medical College, Huazhong University of Science and Technology, Wuhan, China; 2 Hubei Provincial Clinical Research Center for Precision Radiology & Interventional Medicine, Wuhan, China; 3 Hubei Key Laboratory of Molecular Imaging, Wuhan, China

**Keywords:** artificial intelligence, digital health, hospital information system, no-show, online scheduling, radiology, self-service appointment

## Abstract

In recent years, China has accelerated the implementation of precision medicine strategy, integrating accuracy, individualized care and evidence-based decision-making into clinical diagnosis and treatment. Meanwhile, the expansion of smart-hospital and the requirement of Level-5 electronic medical record (EMR) certification have made digital upgrading an indispensable component of radiological services. As a result, self-service appointment is increasingly replacing conventional manual registration and has become an important part of the digital infrastructure of modern radiology departments, with an increasing proportion of administrative pressure, and enhance overall operational performance. Nevertheless, their effectiveness remains restricted by several deficiencies, including limited differentiation among examination workflows, insufficient risk assessment before imaging procedures, inadequate accessibility for older adults, and delayed information exchange between heterogeneous systems; therefore, future improvement should focus on three major directions: (a) AI-supported scheduling tailored to individual patient needs, (b) closed-loop management covering the entire examination process, and (c) coordinated appointment mechanisms involving multiple clinical disciplines, thereby advancing radiology services toward a more refined, intelligent, and patient-centered model.

## 1 Introduction

Chinese radiology departments are currently confronted with substantial workforce constraints, as the existing national pool of imaging professionals remains inadequate and the density of radiologists per million residents is considerably below that reported in many developed nations, leaving medical institutions increasingly unable to accommodate the rising volume of imaging procedures while simultaneously maintaining the expected standard of service (1). Moreover, the combination of limited staffing and escalating image-reading workloads is not confined to China but has become a widely acknowledged concern across healthcare systems worldwide (2, 3), which has encouraged growing interest in introducing self-operated appointment services within hospital imaging departments; nevertheless, because healthcare infrastructure, economic development, and service models vary among regions, the adoption and performance of these systems differ substantially across countries (4). In highly market-oriented developed regions, online self-booking and digital payment have been broadly adopted, with scheduling platforms closely connected to hospital information networks (5–7), whereas other healthcare settings, despite having established radiology booking systems, continue to experience ineffective implementation, including frequent missed appointments, inadequate patient–provider communication, and persistent congestion in examination waiting areas (8, 9).

Propelled by national smart-healthcare strategies and standardized hospital accreditation requirements, including tertiary hospital intelligent-service evaluation and Level-5 EMR qualification (10–12), medical imaging has assumed an irreplaceable role in clinical diagnosis, population-based disease screening, and post-treatment efficacy assessment (3). It now constitutes a core operational module within hospital radiology departments. Radiology services primarily encompass digital radiography (DR), computed tomography (CT), and magnetic resonance imaging (MRI), and are characterized by high daily patient volumes, centralized examination scheduling, compact workflows, and high costs for equipment procurement and maintenance.

In China, major tertiary medical centers generally undertake a considerable number of imaging procedures each day, with university-affiliated and teaching hospitals carrying an especially heavy CT and MRI workload; nevertheless, radiology scheduling historically depended on face-to-face registration and physical queuing, a model repeatedly associated with prolonged waiting, intensive staffing requirements, frequent communication between patients and personnel, and complicated administrative steps (13). Such structural deficiencies diminish convenience and satisfaction for patients while simultaneously restricting improvements in departmental productivity, service digitalization, and the broader information-based capabilities of hospitals.

In recent years, patient-oriented self-service appointment platforms have achieved widespread adoption in radiology departments, with an increasing majority of tertiary hospitals in China now deploying such systems (13, 14). This has fundamentally transformed the traditional manual appointment model and offers patients and families considerable flexibility; in many cases, patients can obtain earlier, faster, and more convenient appointments without being physically present at the hospital, and families can schedule an available time that is convenient for them.

Supported by several access points, such as hospital-based intelligent terminals, WeChat mini-programs, institutional smartphone applications, and online-hospital interfaces, these systems permit patients to independently reserve examinations through a continuously available digital process (15–17); consequently, they have become important tools for reorganizing departmental operations, increasing service productivity, reducing procedural complexity, and improving the overall care experience, while also contributing to intelligent radiology development and more precise institutional management (18). Through interoperable connections among the hospital information system, picture archiving and communication system, and related scheduling modules, hospitals can create an integrated pathway that links remote booking, on-site imaging, and subsequent report delivery (4), whereas, in comparison with counter-based arrangements, digitally enabled self-scheduling removes many temporal and geographical restrictions, reduces dependence on registration personnel, supports more appropriate distribution of imaging resources, and establishes a clearer and more orderly reservation sequence. These advantages provide practical support for radiological services that are increasingly standardized, technology-enabled, and responsive to patient needs.

Concentrating on DR, CT, and MRI as the three principal imaging categories, this review is organized around three objectives: (a) to evaluate the current implementation and multifaceted benefits of patient-directed booking across these examinations, (b) to identify recurring technological and administrative barriers while presenting feasible approaches to improvement, and (c) to anticipate the future development of intelligent appointment platforms in Chinese tertiary hospitals. By addressing these dimensions, the review seeks to offer operational references for system renewal, workflow redesign, and the continued advancement of smart radiology.

Despite its clinical relevance, this topic has not been comprehensively reviewed from a radiology-specific perspective. Existing evidence remains fragmented across informatics, nursing, and general outpatient scheduling, with no prior review systematically comparing self-service appointment platforms across DR, CT, and MRI or integrating implementation status, limitations, optimization strategies, and future directions within a unified framework. This narrative review therefore provides three key contributions: (i) a modality-specific synthesis of current practices and challenges relevant to radiology management; (ii) a critical appraisal of the evidence supporting reported benefits and proposed solutions; and (iii) an evidence-based agenda for future development that clearly distinguishes established findings from expert opinion.

## 2 Eligibility criteria, literature search and screening methods

### 2.1 Eligibility criteria

Studies were considered eligible if they were published between January 2010 and December 2025, written in English or Chinese, and directly relevant to the topic of interest within a radiology-specific context. Eligible publications were required to provide sufficient information to inform at least one of the predefined dimensions of the narrative synthesis: clinical implementation status, differentiated applications, operational challenges, evidence-based optimization strategies, or future development trends. Studies were excluded if their topic was not aligned with the scope of the review, if they did not specifically address radiology or medical imaging practice, or if the full text was unavailable. Conference abstracts, editorials, and publications in languages other than English or Chinese were excluded. Duplicate publications or studies reporting substantially overlapping data were also removed, with the most complete or informative version retained where applicable.

### 2.2 literature search and screening methods

To improve transparency and limit selection bias, a structured but non-protocolized literature-selection process was followed. PubMed, Web of Science, Scopus, China National Knowledge Infrastructure (CNKI), Wanfang Data Knowledge Service Platform (Wanfang Data), and China Science and Technology Journal Database (VIP Database, CQVIP) were searched for the period January 2010 to December 2025, yielding 5675 records in total (PubMed 1250, Web of Science 980, Scopus 1320, CNKI 865, Wanfang Data 742, and VIP database 518). Because the primary search captured relatively few Chinese-language studies, a targeted supplementary search was conducted during revision and identified 13 additional Chinese-language records. Relevant authoritative policy or guidance documents identified through the supplementary targeted search were also considered when they provided substantive information pertinent to the review objectives. The final narrative synthesis therefore comprises 79 records (Figure 1). Two reviewers (P.W.S. and K.K.L.) independently screened and assessed the titles and abstracts for eligibility, and final inclusion was reached based on full-text evaluation. Any disagreement was resolved by discussion until a consensus was determined. Otherwise, a third reviewer (Q.F.) would act as an arbiter. Because much of the literatures are descriptive, the main text explicitly notes study design, sample, and methodological limitations wherever possible, and divergences among studies are highlighted rather than averaged away.

**Figure 1:**
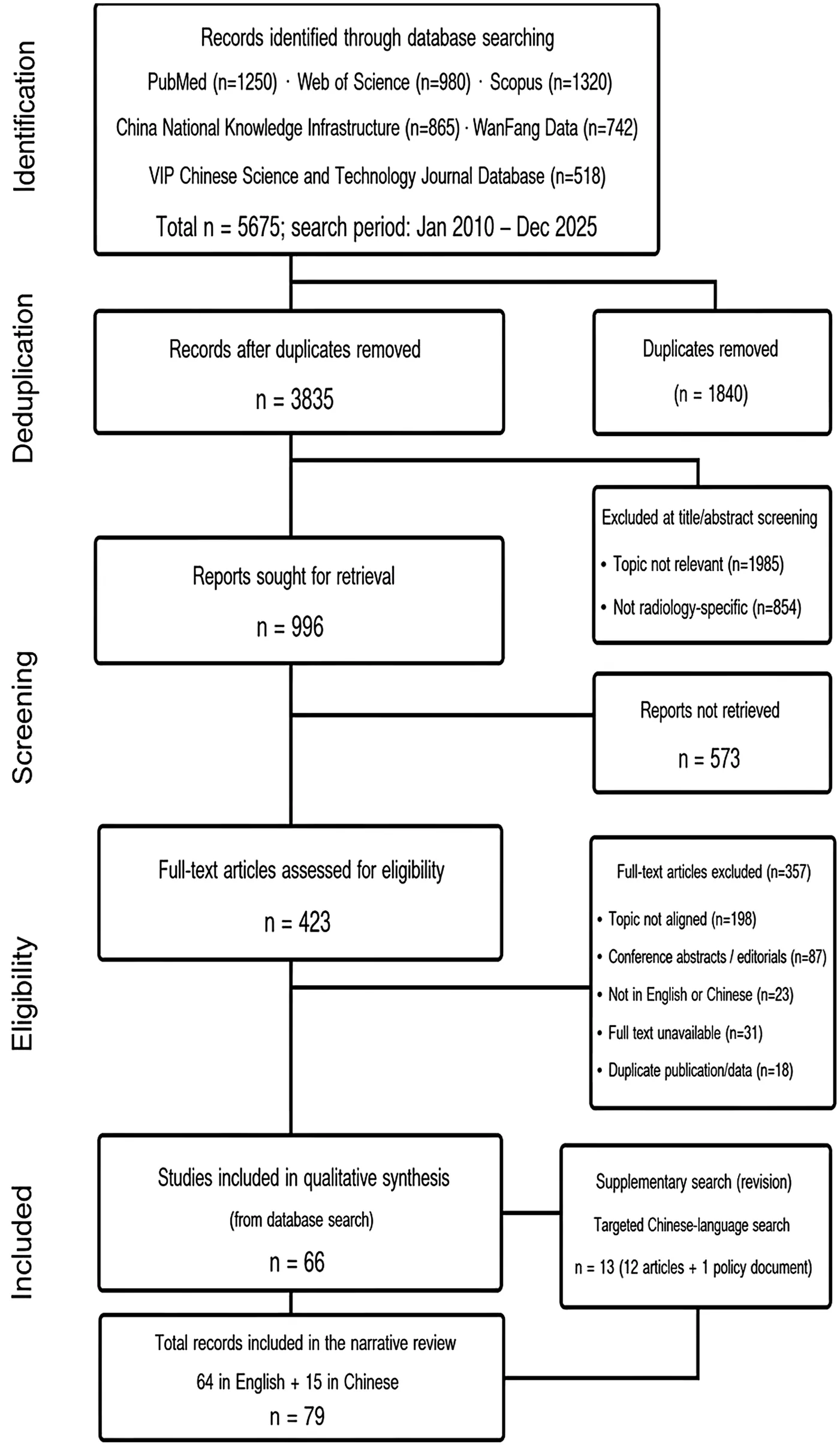
PRISMA flow diagram illustrating systematic review stages: 5675 records identified, reduced to 3835 after duplicates removed, 996 reports sought, 423 full-text articles assessed, 66 studies included from database search, supplemented by 13 additional records, resulting in 79 studies in the narrative review.

## 3 Clinical implementation status of radiological self-service appointment platforms

### 3.1 From manual registration to multi-channel self-scheduling

Initial investigations conducted in China traced the gradual shift from traditional window-based registration to institution-wide appointment systems (13, 19), followed by the growing use of online reservation services in the outpatient departments of major tertiary hospitals (14) later studies further examined the expansion of booking options, including internet platforms, telephone services, and in-person channels, together with the factors influencing patients' selection among these approaches (15, 16), while also documenting the wider transfer of primary healthcare and medical consultation toward web- and smartphone-supported delivery models (17, 18). Recent domestic research has placed greater emphasis on standardized digital reservation procedures intended to facilitate access to specialist consultations and imaging examinations within tertiary institutions (20), with self-operated booking also being introduced into more narrowly defined clinical services, such as nursing care for dry-eye disease (21); meanwhile, healthcare systems in economically developed countries have continued to expand self-scheduling through patient portals, as illustrated by the rapid introduction of combined self-assessment and appointment-booking functions during the COVID-19 period (22), as well as their direct use in diagnostic imaging services (23) and mammographic screening programs (24). Innovations have also increasingly focused on the design and functionality of booking interfaces, including recommendation-algorithm-supported mobile scheduling applications (25) and electronic pre-appointment registration procedures (26). Notably, most of these studies are single-center or descriptive in design, and direct head-to-head comparisons of different booking channels within the same institution are scarce; consequently, the relative effectiveness of each channel for different patient groups remains incompletely characterized.

### 3.2 Differential adoption of self-booking for DR, CT, and MRI

Self-service booking has been broadly introduced in radiology departments of Chinese tertiary hospitals, covering DR, CT, and MRI through multiple online and on-site channels; however, platform functionality and utilization differ across modalities because of variations in procedural complexity, safety risks, and operational demands.

DR is the most suitable and mature modality for independent booking because its protocols are highly standardized, preparation is limited, and adverse-event risk is low. In most hospitals, patients can complete the full process without staff assistance, including selecting an appointment time, confirming the examination, making payment, obtaining a ticket, and checking reservation details, thereby providing an efficient service for most routine cases.

CT services include non-contrast imaging, contrast-enhanced examinations, multiphase scans, and CT angiography (CTA), with many platforms allowing separate booking for routine and emergency procedures while providing basic warnings about contraindications to identify potentially unsuitable patients. Nevertheless, support for technically demanding examinations, particularly multiphase enhancement and CTA, remains limited because these procedures require more complex preparation, risk assessment, and clinical coordination.

MRI self-booking has developed more slowly and remains less common than DR- or CT-based scheduling because MRI involves longer scan times, more contraindications, and stricter safety requirements. Accordingly, MRI modules usually assess metallic foreign bodies, pacemakers and other implanted devices, incompatible internal materials, and claustrophobia, while also informing patients about examination duration, scanning conditions, and essential precautions; these functions help reduce inappropriate reservations, although formal safety screening and written informed consent are generally still required before the examination (27–29). The cited studies are highly heterogeneous in design, outcome measures, and reporting, ranging from platform usage statistics to patient surveys, which limits quantitative synthesis and prevents robust estimation of adoption rates across institutions.

### 3.3 Multidimensional benefits of self-service appointment

#### 3.3.1 Patient-centered value

By removing temporal and geographical restrictions, self-service platforms enable patients to book, modify, pay for, and review appointments through mobile devices, thereby reducing on-site queues, simplifying care procedures, and alleviating common problems such as difficult booking and prolonged waiting; evidence also indicates that self-scheduling can increase screening-mammography completion (24), while telephone, email, and text reminders reduce missed breast-imaging appointments (30), with telephone reminders showing favorable cost-effectiveness in outpatient MRI services (31).

#### 3.3.2 Departmental efficiency

By transferring registration, scheduling, and payment from manual counters to digital platforms, self-service systems reduce staff burden and operational errors, distribute patient flow more evenly, and improve equipment use; intelligent radiology platforms have been associated with greater workflow efficiency and higher satisfaction (32), while optimized MRI templates (33), improved outpatient CT access (34), and interventions promoting timely pediatric cardiac MRI starts (35) further demonstrate the contribution of scheduling design to service capacity.

#### 3.3.3 Patient experience

Satisfaction in radiology is strongly influenced by care quality, comfort, accessibility, and service processes (5), including the experience of breast-imaging patients (36) and the clarity of appointment education (37); studies of online hospital booking, particularly among older adults (38), further show that perceived waiting duration and reservation difficulty remain important determinants of experience (39).

#### 3.3.4 Hospital informatization

Widespread use of self-service booking supports the digital, standardized, and regulated management of radiology services by connecting hospital information system (HIS), picture archiving and communication system (PACS), billing platforms, and nursing workstations, while also contributing to smart-hospital development and compliance with Level-5 EMR assessment requirements (10–12). It should also be emphasized that most reported benefits derive from cross-sectional surveys or before–after evaluations rather than controlled longitudinal studies, so causal attribution of these improvements remains uncertain.

### 3.4 Scheduling optimization and decision support

A broad body of operations research supports the design of radiology booking systems, including fairness-oriented multi-objective scheduling with metaheuristic algorithms (40), coordinated planning of patient time slots and technologist shifts (41), computer-assisted radiology scheduling models (42), online reservation for nuclear medicine (43), real-time patient-centered allocation of ultrasound resources (44), and integrated scheduling platforms covering DR, CT and MRI within high-quality public-hospital development frameworks (45–47) (Figure 2).

**Figure 2:**
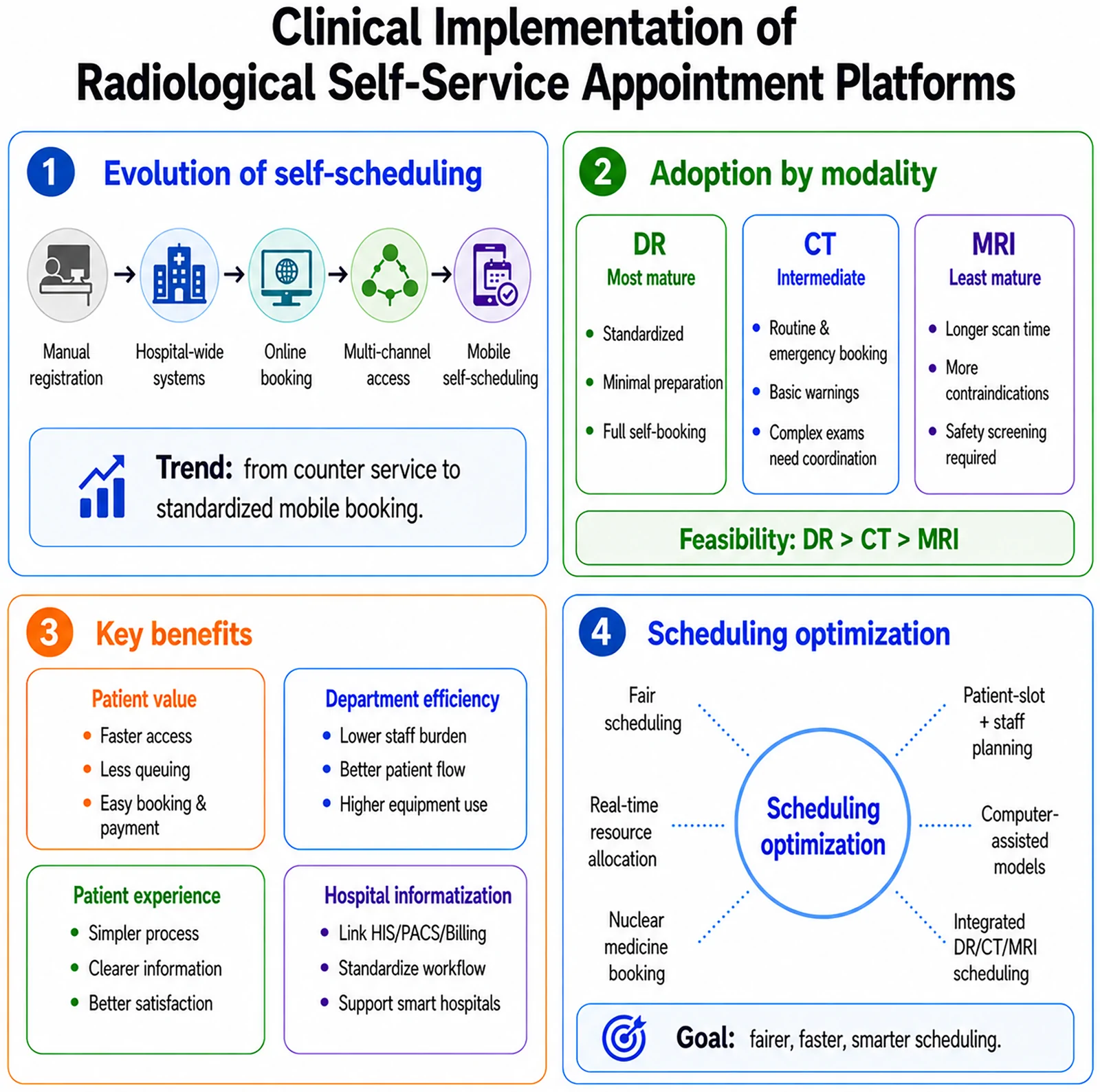
Infographic outlines clinical implementation of radiological self-service appointment platforms. Step one shows the evolution from manual registration to mobile self-scheduling. Step two compares adoption by modality, listing DR as most mature, CT as intermediate, and MRI as least mature. Step three details key benefits such as improved patient value, experience, department efficiency, and hospital informatization. Step four highlights scheduling optimization through fair scheduling, real-time allocation, patient-staff planning, and integrated scheduling, aiming for fairer, faster, and smarter appointments.

## 4 Common deficiencies of current self-service appointment platforms

### 4.1 General functional limitations across modalities

Current self-service platforms often use standardized booking templates that do not distinguish patient age, emergency status, visit type, or consultation context, leaving older adults, non-local patients, and first-time users reliant on staff support; meanwhile, limited guidance on preparation, contraindications, and post-examination precautions may result in delays, unsuccessful examinations, or repeated imaging. Additional weaknesses include restrictive cancellation and rescheduling policies, insufficient credit-based measures to reduce no-shows, and delayed synchronization with HIS and PACS, which can produce inconsistent booking records, payment errors, and abnormal appointment status, thereby waste resources and increase the risk of patient–provider disputes.

### 4.2 No-shows: a persistent operational challenge

Non-attendance remains a major obstacle to efficient radiology scheduling, having long been regarded as a scheduling failure (48) and continuing to generate substantial operational and financial losses (49, 50). Related studies have examined missed appointments through EMR-based regression models (50), large-scale analyses of environmental influences (49), and CT-specific prediction tools (51); they also show that the factors associated with no-shows differ from those leading to active cancellation (52), while sociodemographic characteristics may contribute to unequal attendance patterns, including racial disparities in mammography (53) and differences in missed screening and later completion (54). Evidence from Singapore (7) and qualitative research conducted in Saudi Arabia (9) further clarifies the context-specific causes of non-attendance. Although predictive models achieve promising discrimination in their derivation samples, most have been developed and internally validated on single-institution datasets; external validation and prospective evaluation in Chinese radiology settings are still lacking (55), and reported model performance should therefore be interpreted cautiously.

### 4.3 Modality-specific limitations

Modality-specific limitations remain evident: (a) DR booking categories are often too broad, making it difficult for patients without medical knowledge to identify the appropriate body region and increasing invalid bookings or repeated examinations; (b) CT platforms may not clearly differentiate plain, contrast-enhanced, and CTA protocols, while screening for contrast allergy and renal impairment is frequently inadequate, creating potential safety risks; and (c) MRI systems generally have the weakest safety controls, as optional text-based contraindication prompts can be easily overlooked, and inaccurate estimates of examination duration may disrupt patient planning and aggravate congestion in waiting areas. Empirical studies that directly link specific platform-design features to these modality-specific failures remain sparse; most conclusions are based on institutional experience rather than systematic root-cause analyses of booking and patient-flow data.

### 4.4 Insufficient human-centered design and equity gaps

Insufficient age-friendly design remains common, as small text, low visual contrast, complicated procedures, and limited voice guidance or real-time support hinder independent booking among older adults with impaired vision or low digital literacy (38, 56), infants, patients with renal dysfunction, and individuals prone to allergic reactions. In addition, few studies examine the interaction between platform usability and patient characteristics such as age, literacy, and language; understanding these cross-effects is essential for designing genuinely equitable systems. More broadly, digital scheduling may widen existing inequalities by creating additional access barriers, influencing waiting times, and limiting equitable service use (23, 57) evidence from pediatric imaging, telemedicine for older populations, and health-technology governance therefore emphasizes inclusive development, co-design, and graded accreditation (58–61). Many systems also lack emergency fast-track pathways and risk-stratified guidance for vulnerable groups, including pregnant women, infants, patients with renal dysfunction, and individuals prone to allergic reactions.

### 4.5 System stability, capacity, and integrity

Platform reliability and resource coordination remain major concerns, as peak-period congestion may cause slow responses, failed loading, interrupted reservations, or payment errors without timely alerts or contingency support; meanwhile, inadequate demand allocation often concentrates bookings in morning sessions, leaving later slots underused, and real-time online assistance is still unavailable in many hospitals. System performance is further constrained by limited upstream capacity, including radiologist shortages (1), reporting backlogs (2), and structural barriers to imaging access (62, 63), indicating that digital booking alone cannot eliminate delays without simultaneous workforce expansion and schedule optimization (33, 34). Additional challenges include illegal slot reselling, which has prompted user-profile-based scalper detection (64), and poor alignment with patient preferences, since evidence shows that users value different appointment attributes and do not universally favor selecting all booking details or providers independently (6, 65).

## 5 Evidence-based optimization strategies

### 5.1 Stratified workflow optimization by modality

Experience from major tertiary hospitals suggests that standardized booking templates should be replaced with modality-specific pathways tailored to the complexity and risk profiles of DR, CT, and MRI (41); examination categories and anatomical sites should be more precisely defined, intelligent matching with physician orders should guide item selection, and unnecessary procedures should be reduced according to visit type, urgency, age group, and whether the patient is attending for the first time or follow-up.

### 5.2 Strengthened risk screening and visual health education

Modality-specific mandatory screening should be completed before booking, including contrast-allergy assessment for enhanced CT, checks for metal implants and claustrophobia before MRI, and contraindication evaluation for vulnerable populations; itemized verification and compulsory confirmation should prevent submission when risk assessment is incomplete. Concise educational materials, such as images, short videos, and visual pathways, should provide examination-specific guidance before, during, and after imaging to improve patient preparedness and reduce delays or repeated procedures (37).

### 5.3 Flexible rescheduling/cancellation and resource control

A flexible time-limit mechanism should distinguish general from special patients and routine from emergency examinations, with a green channel for urgent rescheduling or cancellation. A patient appointment credit-evaluation system and no-show control mechanism should impose tiered restrictions on repeat or malicious cancellations, while dynamic release of slots, idle-period reminders, and automatic release of unpaid slots can revitalize idle resources and improve scheduling order.

### 5.4 Human-centered upgrades for vulnerable groups

Age-friendly services should be comprehensively upgraded-larger fonts, stronger contrast, simplified pages, voice broadcast and control, one-click appointment, and manual-assistance calls-while exclusive priority channels and guided booking should be opened for emergency patients, children, pregnant women, and people with disabilities (38, 56, 59). A disease-specific contraindication database can automatically match appointments to patients' conditions and provide tailored guidance and risk alerts.

### 5.5 Cross-system integration and stability reinforcement

Interoperability among self-service platforms, HIS, PACS, laboratory information system, payment modules, and clinical workstations should support real-time exchange of patient data, payment records, booking status, examination information, and reports, thereby reducing online–offline delays and data inconsistencies (4, 65, 66). Peak-hour stability should be strengthened through server and architecture upgrades, while automated warnings, traceable operation logs, and manual backup channels provide continuity during system failures (Figure 3).

**Figure 3:**
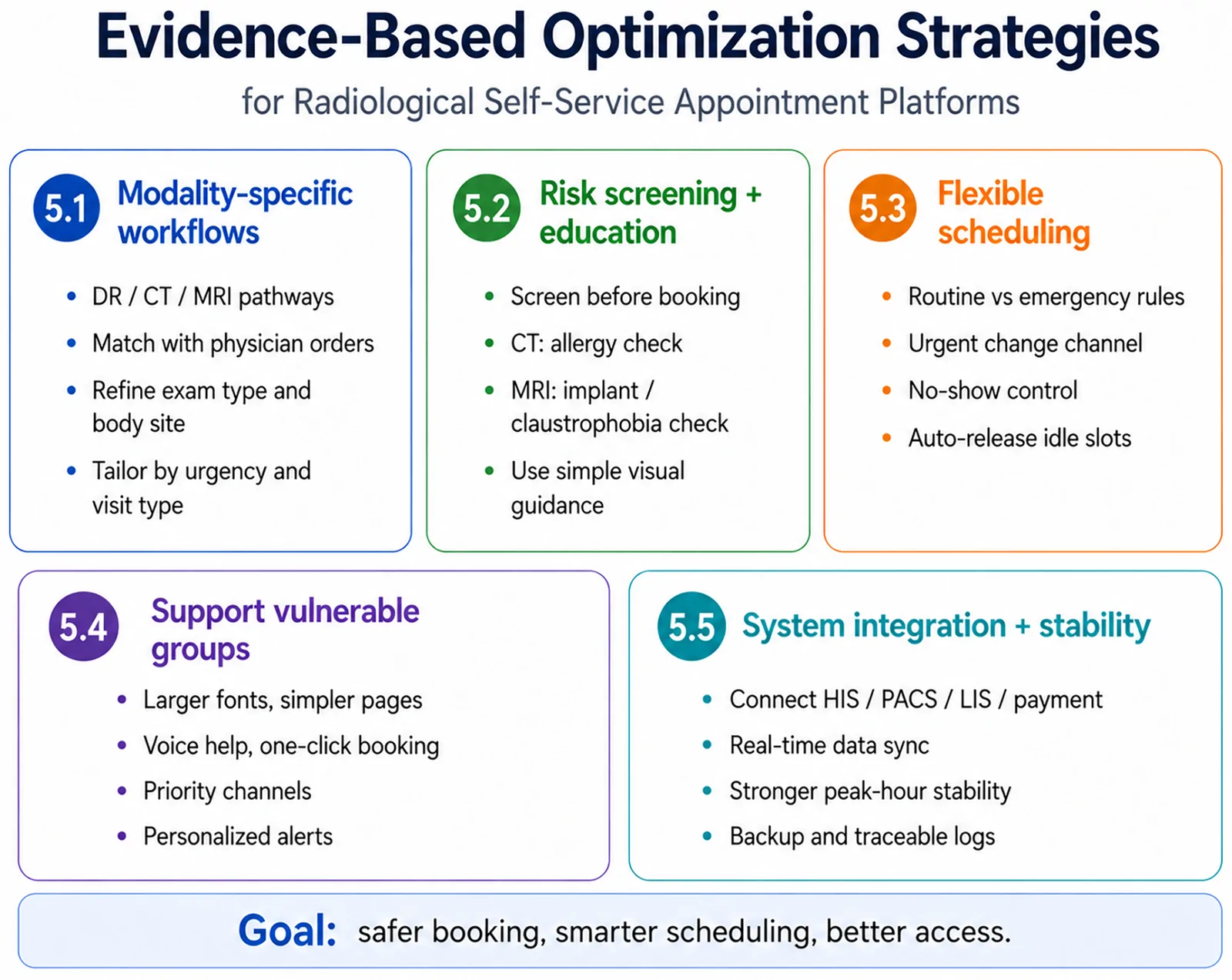
“Evidence-based optimization strategies for radiological self-service appointment platforms” presents five strategies: modality-specific workflows, risk screening and education, flexible scheduling, support for vulnerable groups, and system integration and stability, each with specific action points, aiming to improve booking safety, scheduling, and access.

## 6 Future development trends

### 6.1 AI-driven, intelligent, and personalized reservation

Artificial intelligence (AI) represents a major development direction for radiology self-service systems, enabling automatic retrieval of clinical records and physician orders, verification of examination type, anatomical site, and protocol, and identification of inappropriate requests. Predictive models have been used to estimate outpatient MRI non-attendance (67) and pediatric imaging no-shows or prolonged waits through large language model, regression, and tree-based methods (68), although potential methodological bias requires careful validation (69); meanwhile, broader advances in AI-assisted radiology, including operational applications (70), generative tools (71), intelligent request routing (72), automated report drafting (73), and physician-guided active learning (74), may further support risk-aware scheduling and graded management of high-risk patients. However, the evidence base remains immature: the predictive models cited were mostly developed on retrospective, single-center datasets, and methodological pitfalls—including data leakage, class imbalance, and optimistic evaluation metrics—can inflate reported performance, as cautioned by Fussell and colleagues (69), and these limitations must be addressed before AI-assisted scheduling can be recommended for routine practice. Implementation barriers—such as clinician workload, staff training, integration costs, and resistance to change—are rarely examined in the current literature; although whole-process medical-technology booking platforms have demonstrated improvements in the “daily clearance” of imaging examinations in Chinese tertiary hospitals (27), rigorous controlled evaluations of such closed-loop pathways remain scarce, and future work should therefore assess both effectiveness and feasibility.

### 6.2 Refinement: a full-process closed-loop service system

Future radiology platforms should evolve from isolated booking tools into integrated care pathways linking pre-examination scheduling, on-site imaging support, and post-examination follow-up, with modality-specific services for DR, CT, and MRI; intelligent triage may ease anxiety associated with prolonged waits (75), while synchronized access across mobile programs, self-service terminals, and clinician interfaces can strengthen two-way coordination by allowing staff to monitor appointments and assist with rescheduling or priority access. Evidence on personalized and scenario-based service arrangements remains largely anecdotal or derived from usability studies in other care settings; although an expert consensus on internet-hospital reception services (56) provides practical guidance for older and vulnerable users, controlled evaluations of accompanied, pre-sedation, and accessible booking functions in radiology are still needed before their benefits can be generalized.

### 6.3 Humanization: scenario-based and personalized services

Future services should shift from uniform workflows toward personalized, scenario-specific arrangements, including accompanied, pre-sedation, extended-time, and accessible appointments for children with poor cooperation, older adults, patients with claustrophobia, and people with disabilities. Simplified and multilingual interfaces should improve usability for non-local, international, and digitally inexperienced users (6, 37), while AI-based scheduling should also reflect staff preferences to promote practical acceptance (76). Although performance-appraisal results of Chinese tertiary public hospitals are increasingly used for quality benchmarking (77), quantitative reports linking dashboard-based management indicators to measurable service improvements remain scarce; future studies should track these indicators over time and relate them to operational and clinical outcomes.

### 6.4 Digitalization: smart, refined department management

Big-data analysis can integrate longitudinal booking records to identify demand peaks, equipment use, missed appointments, and repeated examinations, while visual dashboards support staffing, resource allocation, and workflow decisions; indicators such as booking accuracy, on-time performance, and patient satisfaction may also be incorporated into departmental quality assessment (78, 79). Existing evidence on cross-departmental coordinated scheduling is largely derived from operations-research models or single-institution pilots rather than large-scale implementation studies; although workflow redesigns such as fairness- and efficiency-oriented report-distribution processes in radiology (66) have demonstrated single-center gains, the transferability of these benefits to routine practice has yet to be demonstrated.

### 6.5 Collaboration: a multidisciplinary integrated-care model

Future platforms should connect previously separate departments through bidirectional scheduling, enabling clinicians to book, prioritize, or modify examinations directly; coordinated appointment management across radiology, laboratory, ultrasound, and surgery can support one-stop scheduling, reduce time conflicts, and improve overall service efficiency (41, 44, 45).

## 7 Conclusion

Amid the rapid expansion of smart healthcare in China and worldwide, self-service radiology booking has become essential digital infrastructure for tertiary hospitals, supporting DR, CT, and MRI while simplifying patient pathways, reducing staff burden, improving operational efficiency, enhancing service experience, and promoting smart-hospital development. Nevertheless, current systems remain limited by insufficient workflow refinement, weak support for vulnerable populations, unstable performance, and incomplete management mechanisms; these deficiencies may be addressed through modality-specific process optimization, stronger risk screening and patient education, improved cancellation and rescheduling rules, accessible services, cross-system interoperability, and technical reinforcement. With further integration of AI and big-data technologies, future platforms are expected to provide precise scheduling, full-cycle service management, personalized scenario-based functions, data-driven departmental governance, and multidisciplinary coordination, thereby supporting more standardized, efficient, intelligent, and patient-centered radiology services.

To move beyond descriptive summary, we propose a simple conceptual framework—the Modality–Risk–Population (MRP) Framework—that organizes the evidence and recommendations presented above into an actionable structure. The framework rests on three interacting axes: (i) modality (DR, CT, and MRI), which determines workflow standardization, safety requirements, and the depth of booking support needed; (ii) patient risk (e.g., contrast allergy, renal impairment, claustrophobia, and pediatric or pregnant populations), which dictates the stringency of pre-examination screening and the need for human fallback; and (iii) population characteristics (age, digital literacy, language, and social support), which shape usability demands and equity implications. Each deficiency identified in this review—undifferentiated templates, weak MRI safety screening, no-show losses, accessibility gaps, and system instability—can be located at a specific intersection of these axes, and each proposed strategy maps onto the corresponding axis. By making these relationships explicit, the MRP framework provides a testable structure that differentiates this review from purely descriptive accounts and offers administrators a practical heuristic for prioritizing interventions and evaluating platform performance in future research.
